# Environmental surveillance of antimicrobial resistance (AMR), perspectives from a national environmental regulator in 2023

**DOI:** 10.2807/1560-7917.ES.2023.28.11.2200367

**Published:** 2023-03-16

**Authors:** Alwyn Hart, Jonathan Warren, Helen Wilkinson, Wiebke Schmidt

**Affiliations:** 1 Environment Agency, Horizon House, Deanery Road, Bristol, United Kingdom

**Keywords:** Antimicrobial resistance, AMR, Environment, Surveillance, Monitoring, Method, One Health

## Abstract

The development, and in some cases increasing prevalence, of resistance to antimicrobials used in clinical and veterinary settings has long been recognised. In recent years, the concept of ‘One Health’ has added recognition of the role that the environment plays in health protection along with the need for protection of the health of the environment itself. Organisations including the World Health Organization, United Nations Environment Programme, and national governments have identified a need for surveillance of antimicrobial resistance in the environment to sit alongside the surveillance carried out in clinical, veterinary and food sectors. However, having recognised the need for environmental surveillance there are multiple challenges in deciding what this should entail. For example, what pathogens or genes to monitor, who or what we wish to protect and what measures we wish to enable to decrease infection risks. That might include sampling near a source of resistant organisms entering the environment or conversely sampling where the exposure actually occurs. Choices need to be made at both policy and technical levels based on the detailed purposes of surveillance. This paper discusses these issues from the perspective of a national environmental regulator.

## Introduction

Antimicrobials are an essential aspect of modern medicine, with antimicrobial resistance (AMR) being an existential risk for our modern healthcare systems. Although interest in AMR has to date, primarily focussed on clinical resistance to antibiotics, resistance to other antimicrobials (e.g. antifungals) also presents risks to humans, as well as to animal, plant and even environmental health.

The environment can provide sources, and pathways for transmission, of AMR to important receptors including humans [1,2], although the scale is unclear. Nonetheless, the United Nations Environment Programme recognised AMR as one of the most critical pollution issues for the environment [3] and the importance of the dynamic between the environment and AMR has been recognised as part of a multisector ‘One Health’ approach. As a result, reports such as those from O’Neill, the World Health Organization (WHO) and various authors [4-6] have called for surveillance of AMR in environmental settings. Furthermore, WHO has set out minimum recommendations for AMR surveillance which include the environment and food [5]. Central to this is the use of extended spectrum beta-lactamase producing *Escherichia coli* (ESBL-*E. coli*) as an indicator organism and this emphasis on ESBL-*E.coli* has also been recommended by others [6]. Among the global action plans on AMR, the current United Kingdom (UK) National AMR Action Plan includes a specific requirement to establish pilot environmental surveillance at catchment scale to inform future network design [7]. However, government agencies have practical, financial and technical limits to their powers along with a need to be sure that any monitoring data they collect really does provide useful information that can help in protecting people and the environment. Based on the (inter)national calls for environmental AMR surveillance, this paper discusses some of the issues and choices that arise in selecting the most appropriate methods for surveillance for AMR in the environment from the perspective of a government agency that conducts routine environmental monitoring for other health related purposes. Input and advice from clinicians and epidemiologists are needed to ensure that what can be achieved practically in the field delivers relevant information for public health. While some of the issues raised may have global relevance, here we mainly focus on bacterial and fungal resistance for a European context.

## What insights do we want gain from surveillance?

Surveillance for AMR in the environment can provide at least two perspectives:

(i) knowledge of the resistant organisms and resistance genes present in whatever population is contributing to that environment, for example the human and microbial population contributing through sewage discharges,

(ii) knowledge of the resistant organisms that a receptor may encounter through exposure in that environment.

Surveillance can also provide information on how these may change over time. While these perspectives are complementary, the former is largely retrospective and, in some cases, may provide limited additional information to what can be gleaned clinically. Whereas the latter provides information towards risk assessment for potential future infections and intervention options. For a national body with powers and duties relating to environmental regulation including monitoring environmental quality and setting or controlling industrial and agricultural discharges into air and water, this second perspective can fit well alongside existing environmental monitoring often driven by European Union Directives and so both protect receptors and help to identify mitigation options where the risk is sufficiently high.

A related insight could come from knowing the concentrations of active antimicrobials present in a sample, as a potential indicator of selection pressures. However, this would need to be used with a suitable understanding of the types and mechanisms of resistance concerned. Whilst high concentrations of a substance might normally indicate potential for selection pressure, low concentrations might indicate resistance based on destruction of the compound rather than absence of pressure.

## What receptors do we hope to protect?

The One Health concept rightly recognises that human, animal and ecosystem health are interlinked and often need to be addressed together. As an example, major crop species may themselves be at risk from increased resistance to antimicrobials, including antifungal and antiparasitic crop protection products (CPPs). Any increase in resistance to CPPs could not only affect plant health, but also play a role in increasing resistance in some human fungal pathogens; moreover targets of antifungal CPPs are generally metabolic features present in both plant and human pathogens, complicating the development of new antifungal drugs, when resistance to existing ones develops. Currently, antifungal resistance remains relatively little studied compared with antibiotic resistance, particularly within the environment [8] yet studies linking agricultural and clinical resistance in *Aspergillus fumigatus* date back several years [9]. Within the UK large quantities of antifungals are used, accounting for 38% of all pesticide treatment on arable farm crops [10]. Notably, CPPs are often applied at effect concentrations i.e. direct application in the environment leading to the potential for enhanced selection pressure for resistance. Similarly, while it would not be the province of environmental surveillance to directly monitor livestock or food products for antimicrobial resistant pathogens, it seems inevitable that some environmental exposure to resistant zoonotic pathogens can occur for both humans and animals [11]. Even for human health, differences in behaviours will mean that individuals may experience very different exposure scenarios. So, we may wish to identify particular groups such as open water swimmers or surfers as key receptors though they represent only a small proportion of the entire human population [12]. Alternatively, for some hazards the entire population may be exposed, for example to airborne fungal spores which are effectively ubiquitous [2,8].

As a result, an early choice for a surveillance is whether to focus on some, or all, of human, animal, or even plant receptors. This will have a strong influence on what is monitored, where, and how. To date, many surveillance proposals seem to focus almost exclusively on human receptors and on aqueous environments, for example monitoring ESBL-*E. coli* [5,6].

## What pathways do we aim to monitor?

Traditional environmental approaches to human health and chemical risk assessment might recognise three main exposure routes: inhalation, ingestion, and direct contact [13]. Translating these to environmentally acquired risks for AMR we might consider pathways of airborne, waterborne, skin contact and vector borne [14]. So far, efforts on environmental AMR have often emphasised waterborne AMR, particularly due to the release of treated and/or untreated human faecal waste into receiving waters. Clearly, there may be a risk of colonisation or infection to anyone who is then sufficiently exposed to those contaminated waters [12].

The choice of which pathways to monitor should be determined by what risks we wish to understand and in turn will determine what risks we are able to understand and perhaps mitigate. It will also determine what risks we are unable to judge. Since the risks of greatest concern may differ from place to place, or change over time, it follows that surveillance targets may also differ across time and space. Figure A provides an overview of some potential risk linkages that surveillance may seek to follow. While these examples are not an exhaustive list, they do demonstrate that substantial differences exist and include differences in the type of microorganism that may be involved. A further complication is that many potential sources of AMR may contribute both resistant organisms into the environment (air, soil or water) and may deliver antimicrobials that provide selection pressure in the same soil and water leading in turn to new resistance at the point of entry.

**Figure:**
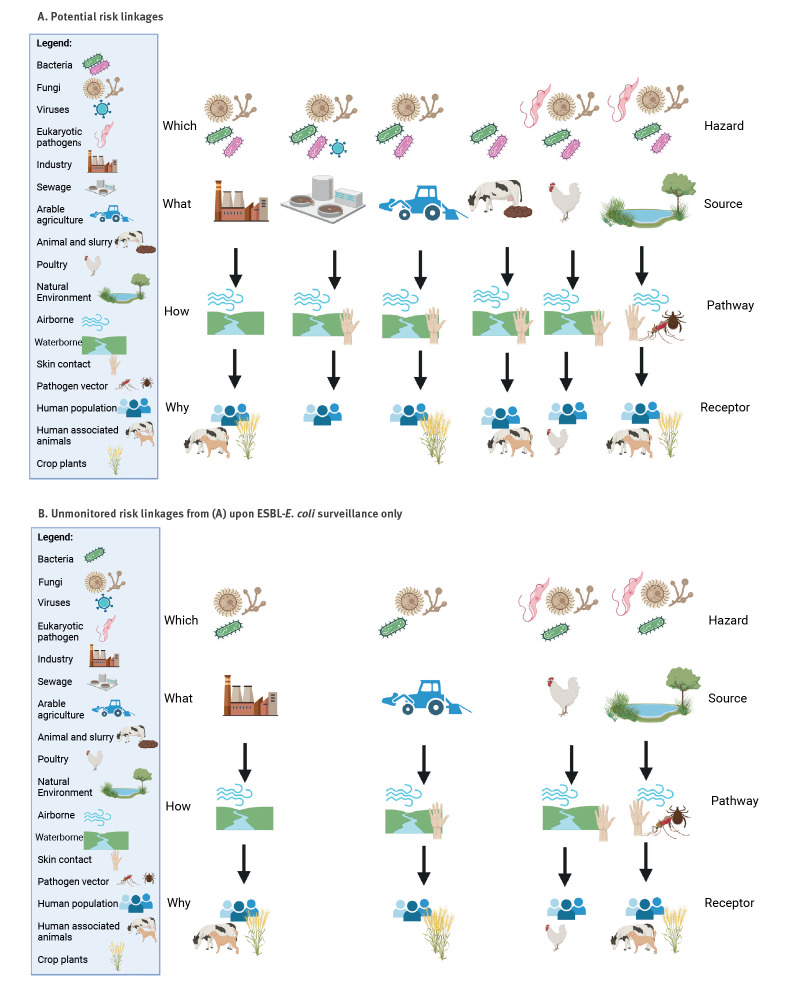
Schematic overview of (A) some potential risk linkages that could inform surveillance and among these, (B) the risk linkages, which will not be monitored by a surveillance scheme to detect only ESBL-*E. coli*, 2023

Created with BioRender.com.

*E. coli*: *Escherichia coli*; ESBL: extended-spectrum beta-lactamases.

In addition, anthropogenic activities are likely to feature strongly in any surveillance programme, for example monitoring may focus on areas where urban wastewater releases occur or where agricultural use of antimicrobials could provide some selection pressure. The urban wastewater itself may contain antimicrobials coming from industrial or clinical production and use as well as resistant organisms from the people concerned. Yet, knowledge of AMR even in pristine natural environments may sometimes also be important and may also provide useful baseline data.

It seems inevitable that whatever surveillance approach is used, not every source–pathway–receptor linkage will be covered. So, a surveillance approach intended to inform about antifungal resistance in airborne spores may have little to tell us about antibiotic resistance from faecal contamination of surface water, or vice versa. Hence, recasting the risk linkages in Figure A based on surveillance of ESBL-*E. coli*, Figure B suggests which linkages may not be served.

## What degree of risk or harm do we want to identify?

Assessment of the impact and consequences of the prevalence of AMR in the environment to human, animal, and/ or plant health is still in its infancy. Does a resistant organism have to be a known pathogen to be of interest? 

While previous research studies have shown that direct exposure routes from the environment to humans exists, e.g. bathing waters and surfing [12], the relationship between exposure, colonisation, and potential infection with AMR-opportunistic pathogens is uncertain and the risk has yet to be quantified. These existing studies have focused on the likelihood of exposure, rather than on the levels of risk of relevant adverse health outcomes associated with an exposure. So key knowledge gaps remain around when, how, and what are the risks or impacts from the carriage of resistant commensal bacteria acquired from the environment. Thus, there is a need for more data on exposure pathways as well as potential relationships for various health outcomes.

Incorporating the assessment of temporal variability of AMR prevalence within a surveillance programme, can provide information on how risk to humans, animals, plants may change with prevailing conditions.

## What mitigation options do we expect to enable?

Larsson et al. [15] identified multiple knowledge gaps on environmental AMR including the question: *“What technological, social, economic, and behavioural interventions are effective to mitigate the emergence and spread of antibiotic resistance via the environment?”*

In veterinary and clinical settings, the obvious mitigation options have been enhanced cleansing and disinfection and decreasing antimicrobial use to limit the selection pressure in favour of resistance [16]. Disinfection of discharges before release into the environment could certainly be a valuable mitigation in tackling environmental AMR, but disinfection within the wider environment is unlikely to be possible except in rare cases. Furthermore, in some cases the treatment methods may even promote the selection for resistance in bacteria [17], while for other cases existing technologies may either be too expensive or impractical to apply. For surveillance data to be useful in targeting disinfection efforts it will need to be capable of identifying hotspots (or hot moments) of AMR burden, and some source apportionment. The source apportionment evidence need not be from the same surveillance analytical method, but could include others such as visual observation, use of chemical markers or microbial source tracking, and integrate with wider environmental monitoring schemes [18].

For interventions that aim to decrease the selection pressure within the environment, surveillance based on chemical analysis may be more relevant than microbiological methods. Over recent years, several approaches have been proposed to determine ‘Minimum Selection Concentrations’ (MSCs) for antimicrobial substances [19,20]. MSCs can then be used to inform policy and regulatory controls where appropriate. For example, in setting discharge limits or application controls for release of substances into the open environment. However, the range of substances for which MSCs are available is still small and the methods in use are still developing, with most of the focus to date having been on antibiotics.

## Conclusions

The need for surveillance of AMR in environmental settings has been well established and some basic minimum qualities proposed such as the WHO Tricycle [5]. However, the detailed design of this surveillance should be driven by accepted environmental risk assessment approaches. Not every location or timeframe will benefit from the same surveillance approach, and much will depend on the agricultural, ecological, veterinary, or public health policy objectives that are being served at that time and in that location. Nonetheless, there are likely to be very similar needs and drivers across most European countries and a joined-up approach would facilitate data sharing and collaborative responses.

It is important that managers of surveillance networks should communicate exactly what their network is intended to provide, what surveillance targets are chosen and just as importantly, what they have left out and why.

Ideally, data that are acquired on environmental AMR should be capable of being recorded and interpreted alongside human and veterinary data, but if we want to understand the risks posed it is equally important that they can be interpreted alongside other environmental data. Existing environmental monitoring networks such as those that report on air or water quality have developed over many years, may contain large numbers of sample sites and often aim to protect human health from anthropogenic pollutants. Although they largely focus on chemicals these networks can present opportunities for cost sharing in sampling and practical considerations, such as site access. They will also provide important context information to help understand AMR surveillance results by reporting on known chemical pollutants and even basic hydrology and meteorology.

In conclusion, environmental AMR surveillance will need to have a clear focus and justify both the risk linkages that they cover and those linkages that they do not. The limitations and uncertainties of any surveillance should be made clear to policymakers both at the network design stage and whenever the resulting data are presented. The technical choices of what to look for and how to do it require human, veterinary, and environmental specialists to work together to deliver relevant and comprehensible data. Antifungal resistance seems to the authors to be largely overlooked in some current proposed surveillance schemes. While for now this is perhaps unsurprising, it is the authors view that surveillance should be capable of looking forward providing a baseline to determine change.
